# Aortic Root Remodeling as an Indicator for Diastolic Dysfunction and Normative Ranges in Asians: Comparison and Validation with Multidetector Computed Tomography

**DOI:** 10.3390/diagnostics10090712

**Published:** 2020-09-18

**Authors:** Lawrence Yu-min Liu, Chun-Ho Yun, Jen-Yuan Kuo, Yau-Huei Lai, Kuo-Tzu Sung, Po-Jung Yuan, Jui-Peng Tsai, Wen-Hung Huang, Yueh-Hung Lin, Ta-Chuan Hung, Ying-Ju Chen, Cheng-Huang Su, Cheng-Ting Tsai, Hung-I Yeh, Chung-Lieh Hung

**Affiliations:** 1 Department of Medicine, Mackay Medical College, New Taipei City 25245, Taiwan; drlawrenceliu@gmail.com (L.Y.-m.L.); med202657@gmail.com (C.-H.Y.); jykuo5813@gmail.com (J.-Y.K.); garak1109@mmh.org.tw (Y.-H.L.); 8905012@gmail.com (K.-T.S.); 4011@mmh.org.tw (P.-J.Y.); amanda1015h2o@yahoo.com.tw (J.-P.T.); 5819.5819@mmh.org.tw (W.-H.H.); 6258.6258@mmh.org.tw (Y.-H.L.); hung0787@ms67.hinet.net (T.-C.H.); chsu007@gmail.com (C.-H.S.); hiyeh@mmh.org.tw (H.-I.Y.); 2 Division of Cardiology, Department of Internal Medicine, Hsinchu MacKay Memorial Hospital, Hsinchu City 30071, Taiwan; 3 MacKay Junior College of Medicine, Nursing, and Management, Taipei City 11260, Taiwan; 4 Department of Radiology, MacKay Memorial Hospital, Zhongshan North Road, Taipei City 10449, Taiwan; 5 Division of Cardiology, Department of Internal Medicine, MacKay Memorial Hospital, Zhongshan North Road, Taipei City 10449, Taiwan; 6 Telehealth Center, MacKay Memorial Hospital, Zhongshan North Road, Taipei City 10449, Taiwan; ying-ju@mmh.org.tw; 7 Institute of Biomedical Sciences, Mackay Medical College, New Taipei City 25245, Taiwan

**Keywords:** aortic root diameter, heart failure, diastolic indices, multidetector computed tomography (MDCT), N-terminal pro-brain B-type natriuretic peptide (Nt-ProBNP)

## Abstract

Background: The aortic root diameter (AoD) has been shown to be a marker of cardiovascular risk and heart failure (HF). Data regarding the normal reference ranges in Asians and their correlates with diastolic dysfunction using contemporary guidelines remain largely unexplored. Methods: Among 5343 consecutive population-based asymptomatic Asians with echocardiography evaluations for aortic root diameter (without/with indexing, presented as AoD/AoDi) were related to cardiac structure/function and N-terminal pro-brain B-type natriuretic peptide (Nt-ProBNP), with 245 participants compared with multidetector computed tomography (MDCT)-based aortic root geometry. Results: Advanced age, hypertension, higher diastolic blood pressure, and lower body fat all contributed to greater AoD/AoDi. The highest correlation between echo-based aortic diameter and the MDCT-derived measures was found at the level of the aortic sinuses of Valsalva (r = 0.80, *p* < 0.001). Age- and sex-stratified normative ranges of AoD/AoDi were provided in 3646 healthy participants. Multivariate linear regressions showed that AoDi was associated with a higher NT-proBNP, more unfavorable left ventricular (LV) remodeling, worsened LV systolic annular velocity (TDI-s′), a higher probability of presenting with LV hypertrophy, and abnormal LV diastolic indices except tricuspid regurgitation velocity by contemporary diastolic dysfunction (DD) criteria (all *p* < 0.05). AoDi superimposed on key clinical variables significantly expanded C-statistic from 0.71 to 0.84 (*p* for ∆AUROC: < 0.001). These associations were broadly weaker for AoD. Conclusion: In our large asymptomatic Asian population, echocardiography-defined aortic root dilation was associated with aging and hypertension and were correlated modestly with computed tomography measures. A larger indexed aortic diameter appeared to be a useful indicator in identifying baseline abnormal diastolic dysfunction.

## 1. Introduction

The aortic root diameter (AoD) gradually increases with age, which may parallel alterations of left ventricular (LV) geometry and diastolic function [1]. In addition to the senescence process, sex, body size (i.e., obesity), hypertension and ethnicity have all been identified as key determinants affecting aortic root size [2,3]. Several epidemiological studies have shown significant associations between aortic root dilatation and several cardiovascular prognosticators, including left ventricular hypertrophy (LVH) and carotid atherosclerotic burden [4,5]. Recent studies have also demonstrated that the AoD may predict future cardiovascular events and incident heart failure (HF) [6,7].

As it has been proposed that central pulse pressure is primarily determined by both aortic stiffness and aortic root geometry [8], a shorter body size though a greater indexed aortic size in ethnic Asians likely renders this population particularly prone to augmented central hemodynamics from peripheral arterial wave reflections [9]. This may likely explain the observed racial differences in hypertension-related cardiovascular diseases [10]. As an emerging new global HF phenotype, heart failure with preserved ejection fraction (HFpEF) is tightly associated with vascular aging and hypertension [11], a shared common pathophysiology with central aortic stiffness and aortic remodeling [12,13,14]. As aortic root dilatation has been shown to be associated with diastolic dysfunction (DD) as a clinical precursor of HFpEF [15,16], understanding the relationship between aortic root remodeling and DD may provide insights into the pathological mechanistic link to HFpEF development in Asians. On the other hand, aortic root geometry, as a complex three-dimensional (3D) geometry, has led to an unmet need for the clinical validity of echocardiography measurements owing to the rapidly growing volume of advanced therapeutic interventions (e.g., transcatheter aortic valve replacement) [17].

Our current study had two major goals. We aimed to explore the clinical and functional relevance of aortic root diameter by utilizing the contemporary American Society of Echocardiography (ASE) guideline [18] and the N-terminal pro-brain natriuretic peptide (NT-proBNP) level proposed by the European HF Society in a large, asymptomatic Asian population that underwent a cardiovascular survey [19]. We further provided normative reference values of aortic root diameter based on age and sex strata in Asians.

## 2. Methods

### 2.1. Study Subjects

This study analyzed a dataset derived from subjects who underwent an annual cardiovascular survey at MacKay Memorial Hospital between July 2003 and December 2012, a tertiary medical center in Taipei, Taiwan. A total of 11,376 subjects provided detailed baseline demographic information and anthropometric measures (including body size, waist, and body fat composition) and were subjected to comprehensive echocardiography studies. Venous blood samples were drawn for detailed blood cell counts, biochemical testing and circulating biomarkers (i.e., high sensitivity C reactive-protein [hs-CRP] and NT-proBNP). Structured questionnaires were obtained from all participants. Among them, 9058 participants had single visits and baseline demographic information collected with paired echocardiography data. The study setting, design, eligibility and exclusion criteria were previously published elsewhere [20]. We excluded subjects with missing baseline variables, known implanted pacemakers, severe pulmonary hypertension (defined as peak systolic pulmonary artery pressure ≥ 60 mmHg), hypertrophic cardiomyopathy, atrial fibrillation, primary significant valvular heart diseases (aortic or mitral valve), or those with prevalent symptoms of heart failure. The study protocol was approved by the local ethical institutional committee (Mackay Memorial Hospital) for a retrospective data analysis, with a waiver of informed consent from the study participants (IRB: 11MMHIS059, 26 May 2011; 16MMHIS142e, 19 April 2018). To explore the associations of aortic root diameter with a variety of cardiac diastolic parameters, only those subjects that met the criteria were included in the current study.

### 2.2. Echocardiography

All echocardiograms were performed by trained technicians according to standardized protocols. A Philips Sonos 5500 ultrasound device (Philips Ultrasound, Andover, MA, USA) was initially used for all echocardiography scans until January 2009 and was subsequently replaced by a GE system (Vivid i/Vivid 7, Vingmed, Horten, Norway) equipped with a 2–4 MHz transducer. LV wall thickness (of the septal (IVS) and posterior wall (PW)), LV internal chamber diameter (end-diastolic (LVIDd) and systolic (LVIDs)), and derived LV mass (LVM) and LV mass indexed (LVMi) to body surface area (BSA) were all obtained from M-mode echocardiographic measures with the patients in the left decubitus position under two-dimensional echocardiographic guidance as recommended by ASE guidelines [21]. LVH was defined as the LVMi. The AoD was measured from the M-mode tracing as the maximal distance between the leading edge to leading edge (L-L) convention of anterior and posterior aortic root wall at the maximal level of the sinuses of Valsalva, as recommended by the ASE [21]. In our current study, we further indexed the AoD by the BSA (AoDi). Volumetric measurements of the left atrial (LA) (max) and LV volumes (both end-diastolic and end-systolic) were quantified using the biplane method of disk summation (modified Simpson’s method), with LA volume further indexed to the BSA as LAVi. Left ventricular diastolic functions were assessed by Doppler echocardiography, which recorded the velocity of transmitral early (E) and late (A) inflow from the apical transducer position, and the E/A ratio was calculated, with deceleration time and isovolumic relaxation time acquired as routine [18]. Tissue Doppler imaging (TDI) by echocardiography, available in the GE system, measured the peak systolic (TDI-s′) and early diastolic (TDI-e′) mitral annulus velocities at both the septal and lateral mitral annuli with the mean values (average) presented. According to DD criteria recommended by the ASE guidelines [18], parameters that define abnormal diastolic indices include (1) abnormal myocardial e′ defined by TDI-based LV septal e′ velocity < 7 cm/s or LV lateral e′ velocity < 10 cm/sec; (2) ratio of transmitral early (E) to averaged LV e′ velocity (from septal and lateral) >14; (3) indexed LA volume (LAVi) > 34 mL/m^2^; and (4) tricuspid regurgitant velocity (TR velocity) >2.8 m/sec, indicating elevated pulmonary artery pressure and velocity.

### 2.3. Serum NT-proBNP Analysis

High sensitivity C-reactive protein (hs-CRP) was assessed by Elecsys 2010 (Roche Diagnostics GmbH, Mannheim, Germany). NT-proBNP (pg/mL) was available for 4735 study participants (88.6%) using an electrochemiluminescence immunoassay (Roche E170, Roche Diagnostics) in this dataset. Major baseline demographic information, including age, sex distribution, body size, blood pressure and medical history, did not differ significantly between study participants with or without NT-proBNP data. Based on the 2016 European Society of Cardiology (ESC) guidelines for HF [19], an NT-proBNP cut-off higher than 125 pg/mL may successfully rule out underlying HF with a high negative predictive value (>90%) in a nonacute setting and was therefore defined as abnormal NT-proBNP in the current study.

### 2.4. Validation on Echo-Based Morphological Aortic Diameter Measure with MDCT

A total of 245 subjects had multidetector computed tomography (mdCT) data for detailed aortic root/diameter sizing and assessment based on three-dimensional (3D) geometry [22]. These measures included diameter assessment at the aortic annulus (AoA), aortic sinuses of Valsalva (AoSV) and sinotubular junction (AoSJ) from coronal oblique CT angiographic noncontrast images [23]. An additional cross-sectional area of the aortic annulus (AoCsA) from double oblique CT angiographic planes was also obtained from 3D workstation software. These measures were then correlated and compared with the echocardiography-defined aortic root diameter in this study.

### 2.5. Statistical Analysis

Continuous data are shown as the means and standard deviations and were compared using the t test. Categorical data are expressed as frequencies and proportions of occurrence for all subjects and were compared using the chi-square test. By setting different aortic diameter quintile strata (treated as linear variables), we tested the linear trends of clinical and all echocardiography parameters with increasing aortic diameter by using Cuzick’s nonparametric trend test. Both AoD and AoD indexed to body surface area (BSA) (referred to as AoDi) were used in the analyses. Determinants of AoD/AoDi from clinical covariates were chosen by using backward stepwise regression (*p* for removal: 0.05), with BSA not entered in models with AoDi. The associations of AoD (per 10 mm +)/AoDi (per 1 unit +) with NT-proBNP and cardiac structural and diastolic functional indices were analyzed using uni- and multivariate linear regression models by adjusting clinical covariates such as age, sex, body mass index (BMI) (which was not included in models with AoDi as it was already indexed to body size), body fat composition, eGFR, medical history of hypertension (HTN), diabetes (DM), coronary artery disease (CAD), hyperlipidemia medication use, regular exercise and active smoking. By excluding subjects with known hypertension, diabetes, cardiovascular disease, hyperlipidemia, active smoking and renal insufficiency (eGFR < 60 mL/min/1.73 m^2^) in our current population, we provided the reference ranges (mean and standard deviation) of AoD/AoDi across different age categories (<30, 30–40, 40–50, 50–60, 60–70, ≥70 years old) stratified by sex. We further tested the linear associations of increasing age (as a continuous variable) with AoD/AoDi and sex differences.

Areas under the receiver operative characteristic (AUC) curve (C-statistics) were calculated to test the diagnostic accuracy of an enlarged AoD/AoDi in identifying abnormally high Nt-ProBNP and abnormal individual diastolic functional indices according to ASE criteria. Diastolic dysfunction (DD) by ASE criteria was defined as the existence of more than half (>50%) of the abnormal diastolic components. In the current analysis, we only analyzed those with at least three diastolic indices available based on ASE criteria (*n* = 5343) [18].

A two-tailed *p* value less than 0.05 was considered statistically significant. The software packages IBM SPSS version 22.0 (SPSS, Chicago, IL, USA) and STATA 14.2 (StataCorp, College Station, TX, USA) were used to conduct the statistical analyses.

## 3. Results

### 3.1. Baseline Characteristics

Among the 5343 study participants (mean age: 49.0 ± 11.1 years, 1862 (34.9%) women) in the final analysis, the mean AoD and AoDi were 32.0 ± 4.0 mm and 17.1 ± 2.1 mm/m^2^, respectively. Men had a significantly larger AoD than women (33.5 ± 3.6 vs. 29.2 ± 3.3 mm), whereas a slightly smaller AoDi was observed in men than women (17.0 ± 2.0 vs. 17.4 ± 2.2 mm/m^2^, both *p* < 0.001) (Table 1). Subjects with larger aortic root diameters were older, were shorter, had a smaller BMI, were more likely to be male, were active smokers, had slightly higher systolic blood pressure, and had worse renal function (lower eGFR) (all trend *p* < 0.05). Subjects with a larger AoDi were more likely to have prevalent hypertension and diabetes mellitus (both trend *p* < 0.001). Subjects with a larger AoD showed broadly similar trends in baseline demographics except for lipid profiles (more unfavorable lipid profiles), a wider QRS duration, and were more likely to be active smokers and alcohol users (Table S1). In general, men had a larger AoD (33.5 ± 3.6 vs. 29.2 ± 3.3 mm) and a slightly smaller AoDi than women (17.0 ± 2.0 vs. 17.4 ± 2.2 mm/m^2^, both *p* < 0.001).

### 3.2. Correlations of Echocardiography-based Aortic Diameter with CT-based Measures

**Figure 1:**
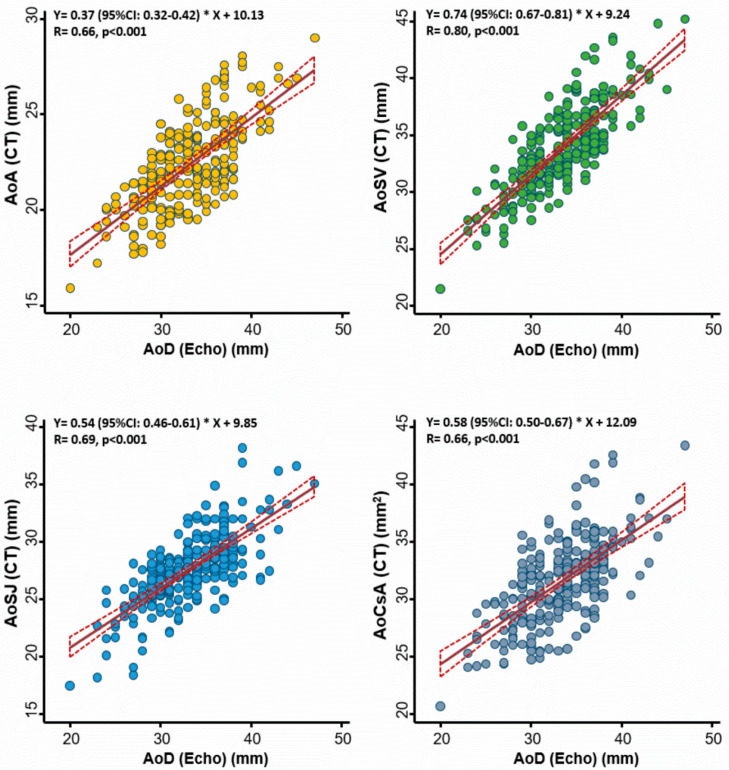
Association between echocardiography- and computed tomography (CT)-defined aortic root diameter at different levels of the aortic root. The best correlation was observed at the level of the aortic sinuses of Valsalva. AoA: aortic annulus; AoSV: aortic sinuses of Valsalva; AoSJ: sinotubular junction; AoCsA: cross-sectional area of the aortic annulus; AoD: aortic root diameter; Echo: echocardiography.

The correlations between echo-based aortic diameter and several CT-based aortic annulus measures among 245 study subjects are displayed in Figure 1. Overall, we observed better correlations between echo-based AoD and CT-based AoSV (r = 0.80), followed by correlations with AoSJ (r = 0.69) and the other two CT-based measures (AoA and AoCsA) (both r = 0.66; all *p* < 0.001).

### 3.3. Clinical Correlates of Aortic Diameter

By backward stepwise-selection regression, advanced age (coef: 1.14 [1.05 to 1.23], per + 10+ years), male sex (coef: 2.30 [1.95 to 2.66]), larger body size in terms of BSA (coef: 7.21 [6.47 to 7.94]), lower body fat component (coef: −0.03), higher diastolic blood pressure (DBP) (coef: 0.03 [0.02 to 0.04]), and the presence of hypertension (coef: 0.49 [0.24 to 0.75]) all contributed to a larger AoD (all *p* < 0.01). Meanwhile, advanced age (coef: 0.78 [0.73 to 0.83], per + 10+ years), female sex (0.69 [0.54 to 0.84]), lower body fat component (coef: −0.09), higher DBP (coef: 0.01 [0.003 to 0.02]), the presence of HTN (coef: 0.21 [0.07 to 0.36]), lower eGFR (coef: 0.05) and active smoking (coef: 0.18, respectively) all contributed to a greater AoDi (all *p* < 0.01). Sex did not modify the association of BSA and AoD (*p* interaction: 0.54).

### 3.4. Age- and Sex-Stratified Normal Reference Ranges of Aortic Diameter

**Figure 2:**
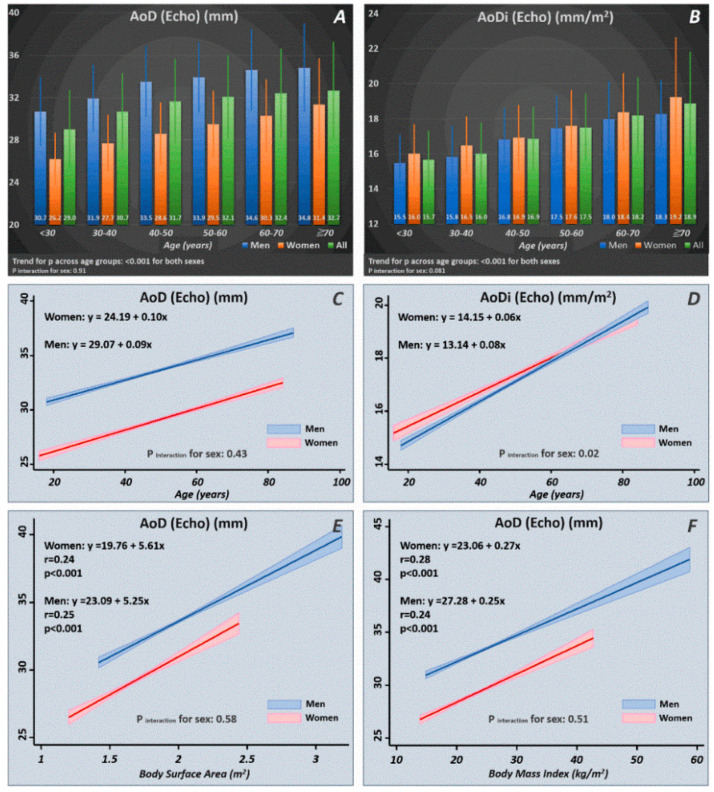
Normal reference ranges of AoD/AoDi by age and sex strata (**A**,**B**) drawn from our healthy participants (*n* = 3646). Linear relationships of AoD/AoDi with increasing age (**C**,**D**) and larger body size in terms of body surface area (BSA) and body mass index (BMI) (**E**,**F**) in men and women. AoD: aortic root diameter; AoDi: indexed aortic root diameter; Echo: echocardiography.

Among the 5343 study participants, we identified 3646 healthy subjects (mean age: 46.8 ± 10.4 years, 1391 women (38.2%)). The distribution of AoD/AoDi in terms of the mean values and standard deviations are displayed in Figure 2. A significant increasing trend of AoD/AoDi was observed with more advanced age for both sexes (*p* for trend: < 0.001 for both). Increasing age as a continuous variable was tightly associated with a larger AoD/AoDi, with men showing a more pronounced AoDi increment with increasing age than women (*p* interaction for sex: 0.02) (Figure 2). A larger BSA and BMI were consistently associated with a larger AoD for both sexes, which showed no sex differences (*p* interaction for sex: NS).

### 3.5. Associations of Aortic Diameter with Cardiac Structure and Function

Multivariate linear regression models showed that both higher AoD and AoDi were independently associated with unfavorable LV remodeling, including greater LV wall thickness, larger LV internal diameter, greater indexed LV mass, greater degree of LV concentricity, larger LV volume, longer isovolumic relaxation time (IVRT), more reversed E/A ratio, worsened LV systolic (TDI-s′) and early relaxation velocities (TDI-e′), and a greater likelihood of presenting with LVH (all *p* < 0.05) (Table 2 and Table S2). A greater AoDi but not AoD was independently associated with a higher NT-proBNP level, larger indexed LA volume and higher LV E/e′ (all *p* < 0.05), whereas a larger AoD was independently associated with a lower left ventricular ejection fraction (LVEF) and more prolonged DT, with these associations being nonsignificant for AoDi.

### 3.6. Associations of Aortic Diameter with Diastolic Dysfunction by ASE Criteria

**Figure 3:**
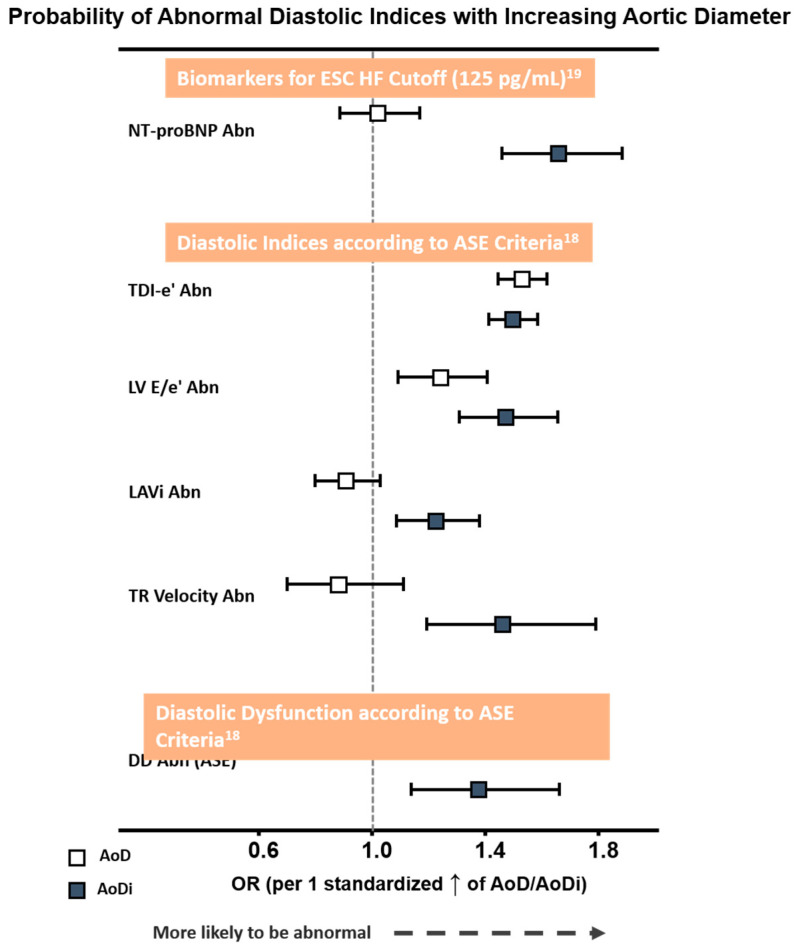
Probability of abnormal NT-proBNP by ESC HF cut-off (>125 pg/mL) and diastolic functional indices proposed by ASE criteria with larger AoD and AoDi. The odds ratio (OR) was 1.44/1.50 for LV septal e′ and lateral e′, both *p* < 0.001. Abbreviations as in Table 2. Abn: abnormality; AoD: aortic root diameter; AoDi: indexed aortic root diameter; E/e′: averaged early mitral inflow E to mitral annulus relaxation velocity e′ ratio; TDI-e′: averaged peak mitral annulus relaxation velocity e′; LAVi: left atrial volume index; Nt-ProBNP: N-terminal pro-brain B-type natriuretic peptide.

Overall, a greater AoDi was more tightly associated with an abnormally high NT-proBNP defined by the ESC HF cut-off (>125 pg/mL) and with each DD component proposed by ASE than AoD (Figure 3). Receiver operating characteristic (ROC) curves generated for AoD/AoDi showed comparable discriminatory performance for abnormal LV e′ (C-statistic: 0.60 for septal, 0.62 for lateral, and 0.61 for averaged LV e′, respectively), with AoDi alone outperforming AoD for discriminating higher NT-proBNP and LV E/e′ (C-statistic: 0.62 and 0.61 for AoDi vs. 0.50 and 0.56 for AoD, respectively, both ∆AUC <0.05). Likewise, AoDi alone yielded better discrimination than AoD for abnormal LAVi and TR velocity (Figure S1) and for DD (C-statistic: 0.58 and 0.50, respectively). A higher AoDi was more likely to be associated with DD defined by the ASE after combining all four diastolic indices (OR: 1.37 (95% CI: 1.14–1.66), *p* = 0.001) but not AoD (OR: 0.98 (95% CI: 0.80–1.21), *p* = 0.87 per 1 standardized increment), with AoDi set at 17.8 mm/m^2^ as the optimal cutoff for underlying ASE-defined DD. While baseline key clinical characters (including age, sex, heart rate, BMI, medical history of hypertension, diabetes, CAD, active smoking and eGFR) exhibited an AUROC of 0.71 (95% CI: 0.65–0.76) in predicting DD, AoDi superimposed on baseline clinical characters further provided significant incremental values in identifying DD (AUROC: 0.84, 95% CI: 0.80–0.89, *p* for ∆AUROC: < 0.001) (Figure S2).

## 4. Discussion

In our large population-based Asian cohort, we demonstrated that along with greater BSA, increasing age, lower body fat fraction, and presence of HTN were markedly associated with larger aortic root size assessed at the level of the sinus of Valsalva. The close relationships between aging, hypertension, diastolic blood pressure and enlarged aortic diameter as indicators of several individual parameters for diastolic dysfunction recommended by ASE perhaps explains in part the more prominent impact of HTN on cardiovascular disease in Asians. We further provided normative reference values for these measures according to our healthy ethnic Asian individuals.

Aortic root diameter and stiffness have been observed to increase with age and are commonly considered normal senescence processes [2,24,25]. This vascular aging phenotype is particularly prominent among Asian patients with hypertension [9], presumably mediated by a continuous loss of elastin content and deposition of interstitial collagen fibrotic replacement. This may result in both degenerative aortic root dilatation and ventricular remodeling leading to HF [1,3,24,26]. Consistent with a prior report, aortic root diameter is strongly affected by age and body size (Figure 2). In accordance with prior studies, we observed that men had larger aortic root diameters [27,28]. Notably, when aortic root diameter was further indexed by BSA, women had a slightly higher aortic root index than men in our Asian population. Degenerative aortic elastic media changes (also termed medial degeneration (MD) of the great arterial vasculature) have been proposed to occur with aging, which likely contribute to aortic root dilation and pathological LV remodeling, thereby resulting in excessive LV mass and ventricular hypertrophy (LVH) [29,30]. Typically, medial degeneration (MD) involves elastic fiber fragmentation and smooth muscle cell loss from cycled damage in the aging aorta or from unfavorable vascular wall hemodynamics [31]. On the other hand, several clinical comorbidities may trigger proinflammatory responses and impaired downstream endothelial nitric oxide (eNO) production complicating both altered vascular arterial properties and aggravated myocardial ischemia, which likely contribute to the pathogenesis of DD or HF (i.e., HFpEF) [32,33]. Although conflicting data exist regarding the true impact of hypertension and systolic blood pressure on aortic root dilation [34,35,36], we did observe a consistent relationship between higher diastolic blood pressure and greater aortic diameter in our Asian population.

Despite the known relationship between AoD and impaired diastolic parameters (i.e., E/A ratio, E and e′) as suggested by Masugata et al. [16], the association of AoD with DD using contemporary ASE guideline recommended criteria has been less studied. Furthermore, it is also unclear whether those participants with increased AoD may demonstrate a correlation BNP level, currently used as a clinical surrogate and predictor for HF according to ESC guidelines and the Framingham Heart Study [37]. As a multifaceted syndrome, traditional LV parameter such as left ventricular ejection fraction (LVEF) may not accurately reflect the extent of myocardial damage underlying heart failure (i.e., HFpEF) [38]. Instead, DD has shown promise in better characterizing LV dysfunction even in HF patients with relatively preserved LV systolic function [15]. AoD has been shown to be related to a future risk of HF among middle-age and older adults [13]; indeed, we observed that a greater indexed aortic diameter in our Asian population was inversely correlated with LV systolic s′ and positively associated with several diastolic functional deteriorations recommended by the ASE, which paralleled a higher NT-proBNP level. Unlike the CARDIAC Study [35], we did not observe associations of increased AoD with impaired LV E/e′, likely explained by the relatively healthy and preclinical status in the current study population and the lack of a longitudinal study design. Given these findings, we suggested that indexed aortic diameter likely serves as a better surrogate reflecting coupled pathological ventricular-arterial degeneration in our ethnic Asian population than the aortic diameter alone, which was in line with a prior report [21].

Recent progress in transcatheter valvular interventions (such as TAVR) has provided new insights into the complex structural aortic root anatomy and functional asymmetry by using advanced imaging or biomechanical approaches [39,40]. Our data showed that the traditionally assessed aortic diameter by transthoracic echocardiography only correlated modestly with those obtained by the CT method, with the best correlation observed with the CT-defined diameter of the aortic sinuses of Valsalva. These results may be partially due to its asymmetrical geometry, a disparity in measurement standards and the challenge of fixing on the same level of aortic root measure when imaging in M-mode during cardiac motion [41]. Our findings indicate and support the concept that no single measure can be uniformly applied when assessing the aortic valve apparatus or geometry [34].

## 5. Limitations

Despite the considerable number of study participants with detailed clinical and echocardiographic parameters, several limitations should be acknowledged. The cross-sectional design of this study and the lack of longitudinal follow-up information may weaken the interpretations of the causal associations of AoD with other conditions. However, the significant correlation with adverse BP-associated LV remodeling and consequent diastolic dysfunction and elevation of the selected biomarker for heart failure mitigate this limitation. The analyzed data were obtained from an asymptomatic population of Asian participants receiving medical health check-ups. Thus, our findings are confounded by selection bias and might not be representative of other ethnic groups.

## 6. Conclusions

Aortic root enlargement is closely associated with increasing age and higher systolic blood pressure and is linked to LV remodeling and diastolic dysfunction in asymptomatic Asians. Our data suggest a shared pathological process between aortic root remodeling and subclinical myocardial damage. We have also provided age- and sex-stratified AoD/AoDi reference values in the Asian population.

## Figures and Tables

**Table 1:** Baseline characteristics of the study population stratified by indexed aortic root diameter.

AoDi Quintiles	1st Quintile	2nd Quintile	3rd Quintile	4th Quintile	5th Quintile	*p* (Trend)
Range	(<15.4 mm/m^2^)	(15.4–16.5 mm/m^2^)	(16.5–17.5 mm/m^2^)	(17.5–18.7 mm/m^2^)	(≥18.7 mm/m^2^)	
Number, *n*	(*n* = 1075)	(*n* = 1061)	(1075)	(*n* = 1067)	(*n* = 1062)	
Age, y	43.6 ± 10.3	46.7 ± 10.4	48.7 ± 10.3	51.1 ± 10.5	56.4 ± 10.5	<0.001
Male sex, %	680 (69.5%)	675 (69.2%)	610 (62.7%)	610 (62.1%)	551 (57.0%)	<0.001
Height, cm	168.9 ± 8.64	167.2 ± 8.12	165.4 ± 7.93	163.7 ± 8.15	161.1 ± 8.44	<0.001
Weight, kg	74.7 ± 14.7	69.3 ± 11.9	66.5 ± 11.3	63.5 ± 10.7	59.9 ± 10.2	<0.001
BMI, kg/m^2^	26.1 ± 4.33	24.7 ± 3.41	24.2 ± 3.15	23.6 ± 3.11	23.0 ± 3.02	<0.001
SBP, mmHg	123.5 ± 17.1	121.3 ± 16.0	122.0 ± 17.1	122.3 ± 17.1	126.0 ± 18.9	0.001
DBP, mmHg	75.9 ± 11.4	75.2 ± 10.6	75.6 ± 10.8	75.6 ± 10.9	76.3 ± 11.3	0.25
HR, per min	75.5 ± 11.1	74.4 ± 10.0	74.4 ± 10.2	74.5 ± 10.4	74.2 ± 9.93	0.02
Fasting glucose, mg/dL	100.8 ± 22.8	99.9 ± 18.2	100.2 ± 20.4	100.2 ± 18.0	103.7 ± 30.1	0.009
Cholesterol, mg/dL	200.0 ± 36.1	200.3 ± 36.1	199.6 ± 35.4	203.6 ± 41.1	202.8 ± 37.2	0.02
Triglyceride, mg/dL	150.1 ± 128.0	138.4 ± 95.8	133.0 ± 98.8	134.3 ± 145.9	126.9 ± 90.7	<0.001
LDL, mg/dL	128.9 ± 33.1	129.2 ± 33.4	128.0 ± 31.9	131.5 ± 34.8	129.7 ± 34.1	0.28
HDL, mg/dL	50.6 ± 14.2	52.1 ± 14.1	53.4 ± 14.8	54.6 ± 14.7	56.4 ± 16.4	<0.001
eGFR, mL/min/1.73 m^2^	90.3 ± 17.0	89.6 ± 16.7	88.8 ± 17.2	88.0 ± 17.8	86.7 ± 18.7	<0.001
QRS duration, ms	90.3 ± 11.1	89.7 ± 10.8	88.9 ± 10.5	88.7 ± 10.4	89.8 ± 13.6	0.12
Hypertension, %	141 (14.4%)	162 (16.6%)	176 (18.1%)	195 (19.8%)	235 (24.3%)	<0.001
Diabetes, %	47 (4.8%)	50 (5.1%)	52 (5.3%)	82 (8.3%)	96 (9.9%)	<0.001
Hyperlipidemia treatment, %	78 (8.0%)	67 (6.9%)	71 (7.3%)	86 (8.7%)	84 (8.7%)	0.23
CAD, %	63 (5.9%)	47 (4.4%)	65 (6.1%)	67 (6.3%)	72 (6.8%)	0.17
Alcohol, %	59 (6.0%)	71 (7.3%)	61 (6.3%)	65 (6.6%)	66 (6.8%)	0.71
Exercise, %	115 (11.8%)	145 (14.9%)	152 (15.6%)	154 (15.7%)	141 (14.6%)	0.07
Active smoking, %	100 (10.2%)	127 (13.0%)	124 (12.7%)	112 (11.4%)	111 (11.5%)	0.77

AoDi: indexed aortic root diameter; BMI: body mass index; SBP: systolic blood pressure; DBP: diastolic blood pressure; HR: heart rate; Hb: hemoglobin; WBC count: circulating white blood cell count; LDL: low-density lipoprotein; HDL: high-density lipoprotein; GPT: glutamic-pyruvate transaminase; eGFR: estimated glomerular filtration rate; CAD: coronary artery disease.

**Table 2:** NT-proBNP level and echocardiographic parameters stratified by indexed aortic root diameter.

AoDi Quintiles	1st Quintile	2nd Quintile	3rd Quintile	4th Quintile	5th Quintile	*p* (Trend)	Pearson’s Correlation	Coef	95% CI	*p* Value
*Biomarker*										
NT-proBNP, pg/mL	33.4 ± 36.0	37.3 ± 46.9	40.9 ± 44.3	45.6 ± 102.5	65.8 ± 180.1	<0.001	0.135	6.50	5.08, 7.92	<0.001
*Cardiac Structure/Function*										
IVS, mm	8.98 ± 1.04	9.03 ± 1.07	9.07 ± 1.05	9.08 ± 1.09	9.24 ± 1.11	<0.001	0.073	0.04	0.02, 0.05	<0.001
LVIDd, mm	46.8 ± 3.51	46.8 ± 3.62	46.5 ± 3.78	46.4 ± 3.60	46.4 ± 3.91	0.002	−0.045	−0.08	−0.12, −0.03	0.002
LVMI, g/m^2^	70.9 ± 12.4	74.3 ± 13.3	76.0 ± 13.4	77.9 ± 14.1	83.1 ± 16.7	<0.001	0.294	2.05	1.86, 2.24	<0.001
RWT	38.5 ± 4.2	38.7 ± 4.3	39.1 ± 4.6	39.3 ± 4.6	39.9 ± 4.9	<0.001	0.11	0.24	0.18, 0.30	<0.001
LVH (%)	7 (0.7%)	8 (2.9%)	21 (2.2%)	36 (3.7%)	99 (10.2%)	<0.001	—	—	—	—
EDVi, mL/m^2^	38.4 ± 5.5	40.0 ± 5.8	40.3 ± 6.2	41.2 ± 6.2	43.0 ± 7.4	<0.001	0.25	0.77	0.68, 0.85	<0.001
LVEF, %	62.2 ± 5.7	62.7 ± 5.2	62.9 ± 5.3	62.8 ± 5.3	62.7 ± 5.6	0.046	0.041	0.11	0.04, 0.18	0.003
DT, ms	196.7 ± 41.3	200.0 ± 40.8	202.1 ± 42.5	204.0 ± 41.7	212.5 ± 45.7	<0.001	0.121	2.47	1.89, 3.04	<0.001
IVRT, ms	85.9 ± 12.3	88.9 ± 13.6	89.5 ± 14.7	89.6 ± 15.5	94.2 ± 17.6	<0.001	0.180	1.30	1.08, 1.53	<0.001
E/A ratio	1.28 ± 0.42	1.24 ± 0.42	1.22 ± 0.42	1.19 ± 0.42	1.08 ± 0.43	<0.001	−0.162	−0.03	−0.04, −0.03	<0.001
TDI-s′ (average), mm	8.42 ± 1.55	8.40 ± 1.54	8.33 ± 1.54	8.19 ± 1.54	7.95 ± 1.60	<0.001	−0.109	−0.08	−0.10, −0.06	<0.001
*Diastolic indices by ASE* [18]										
LAVi, mL/m^2^	16.2 ± 5.08	15.9 ± 5.59	16.4 ± 5.70	16.5 ± 6.20	17.1 ± 6.51	<0.001	0.071	0.20	0.12, 0.28	<0.001
TDI-e′ (average), mm	9.79 ± 2.42	9.57 ± 2.39	9.30 ± 2.43	8.87 ± 2.28	8.16 ± 2.27	<0.001	−0.236	−0.28	−0.31, −0.24	<0.001
E/e′ (average)	7.39 ± 2.22	7.52 ± 2.24	7.75 ± 2.40	8.11 ± 2.55	8.60 ± 2.98	<0.001	0.171	0.21	0.17, 0.24	<0.001
TR velocity, m/sec	17.3 ± 4.85	17.5 ± 5.09	17.7 ± 5.29	17.9 ± 5.55	18.8 ± 5.98	<0.001	0.114	0.29	0.22, 0.37	<0.001

AoDi: indexed aortic root diameter; ASE, American Society of Echocardiography; DT: mitral inflow deceleration time; E: early mitral inflow velocity; TDI-e′: peak mitral annulus relaxation velocity e′; E/A: mitral inflow E/A ratio; E/e′ mean: mean early mitral inflow E to mitral annulus relaxation velocity e′ ratio; IVRT: isovolumic relaxation time; IVS: interventricular septal wall thickness; LA: left atrial/atrium; LAVi: left atrial volume index; LVIDd: LV end-diastolic diameter; LVMI: left ventricular mass index; TDI-s′: peak mitral annulus systolic velocity; TR: tricuspid regurgitation; CI: confidence interval. —: as LVH was a binary data, Coef not calculated.

## Supplementary Materials

The following are available online at https://www.mdpi.com/2075-4418/10/9/712/s1, Figure S1: Receiver Operating Characteristic (ROC) curves for NT-proBNP level and diastolic dysfunction parameters according to criteria recommended by ASE guideline, Figure S2: Receiver Operating Characteristic (ROC) curves for diastolic dysfunction (DD) according to criteria recommended by ASE guideline, Table S1. Baseline characteristics of the study population stratified by aortic root diameter, Table S2. NT-proBNP level and echocardiographic parameters stratified by aortic root diameter.
