# Supplementary material for: Aortic Root Remodeling as an Indicator for Diastolic Dysfunction and Normative Ranges in Asians: Comparison and Validation with Multidetector Computed Tomography
Source: Diagnostics (Basel). 2020 Sep 18;10(9):712. doi: 10.3390/diagnostics10090712 (PMC7555013; doi:10.3390/diagnostics10090712)
Supplement: Supplementary file 1 [file diagnostics-10-00712-s001.pdf]

Supplemental Materials

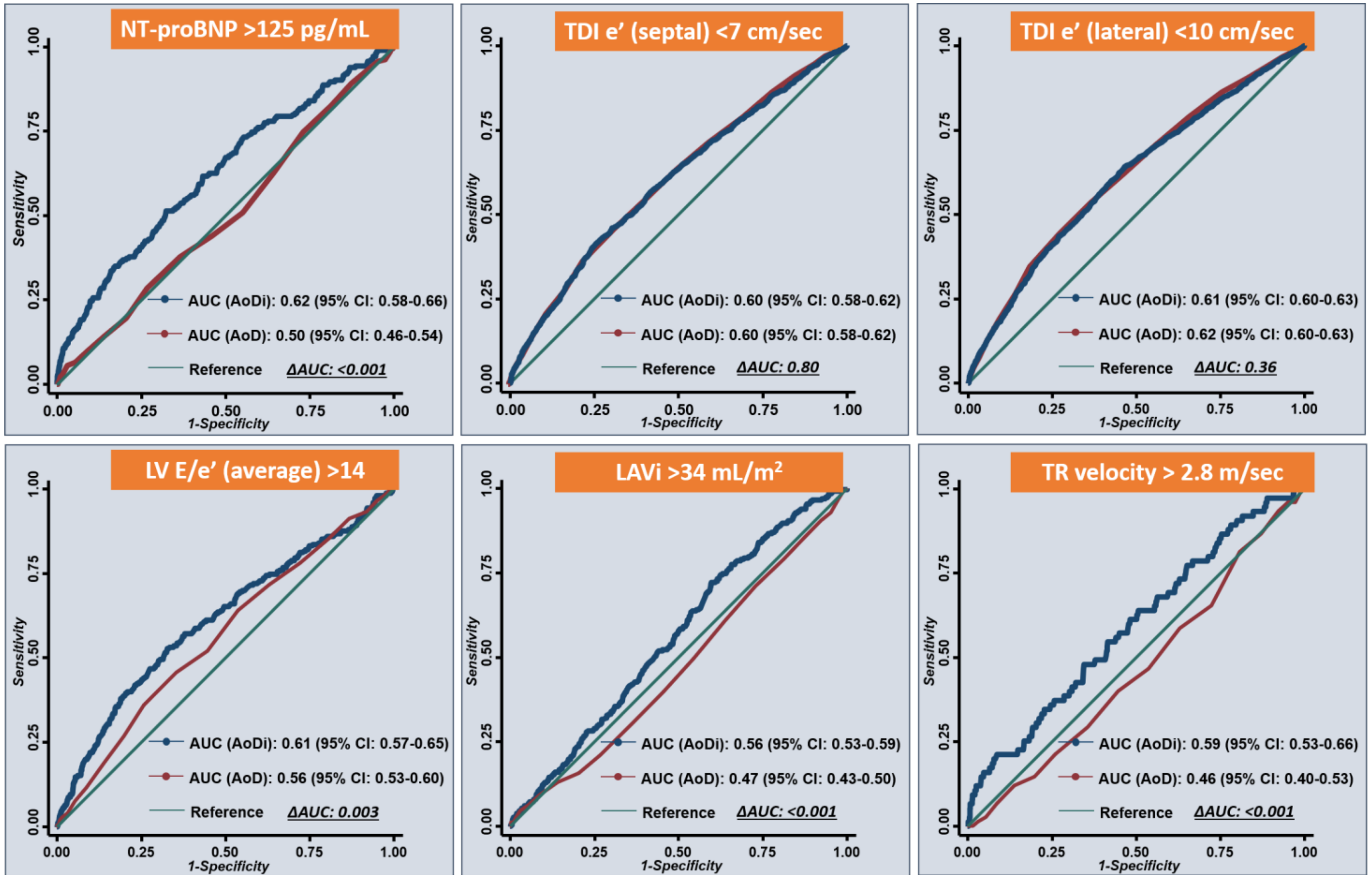

Figure S1. Receiver Operating curves (ROC) for NT-proBNP level and diastolic dysfunction parameters according to criteria recommended by ASE guideline.

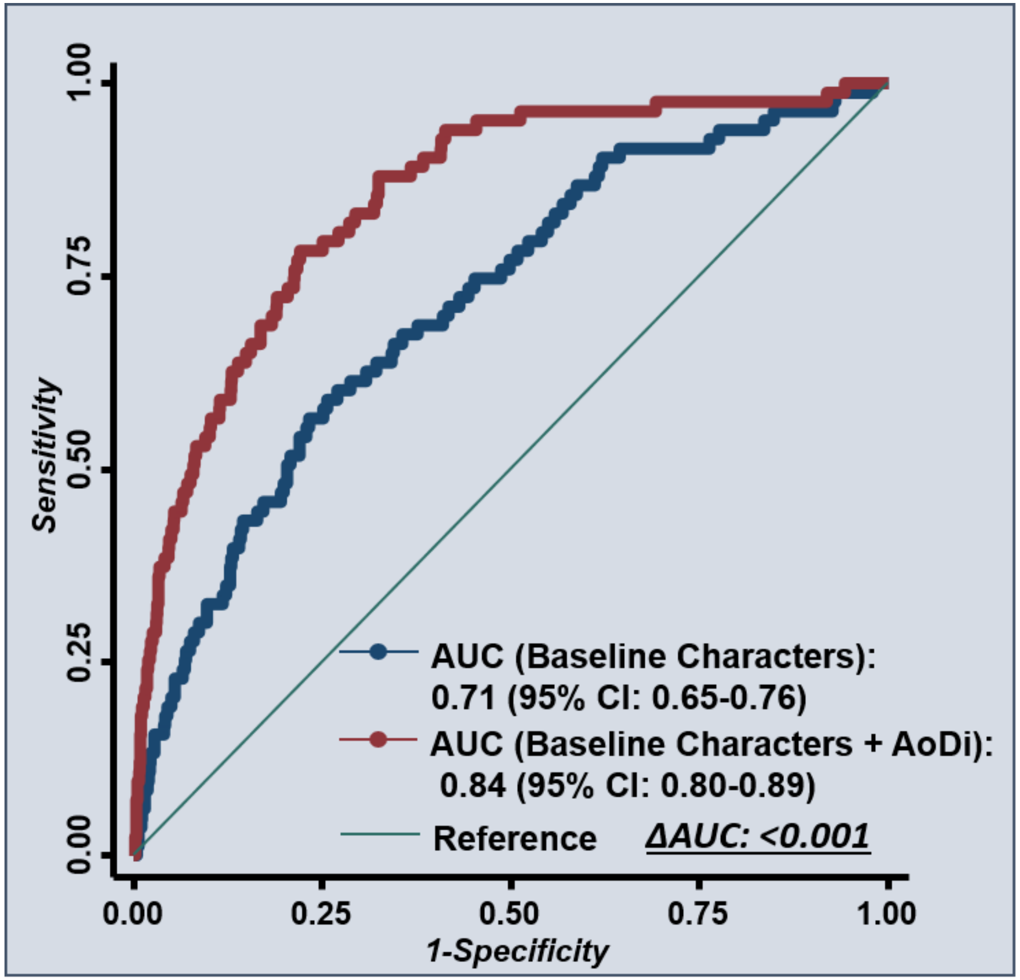

Figure S2. Receiver Operating curves (ROC) for diastolic dysfunction (DD) according to criteria recommended by ASE guideline. Baseline clinical characters included age, sex, heart rate, BMI, medical history of hypertension, diabetes, CAD, active smoking and eGFR.

Table S1. Baseline characteristics of the study population stratified by aortic root diameter.

AoD Quintiles<br>Range	1st Quintile<br>(<30) (*n* = 1481)	2nd Quintile<br>(30–31) (*n* = 982)	3rd Quintile<br>(32–33) (*n* = 968)	4th Quintile<br>(34–36) (*n* = 1161)	5th Quintile)<br>(≧37) (*n* = 748	*p* (Trend)
Age, y	46.6 ± 11.6	48.0 ± 11.1	49.1 ± 10.5	50.0 ± 10.8	52.5 ± 10.5	<0.001
Male sec, %	418 (28.3%)	595 (60.8%)	741 (76.7%)	713 (85.4%)	992 (93.3%)	<0.001
Height, cm	160.3 ± 7.80	164.8 ± 8.31	166.9 ± 8.21	168.4 ± 7.97	169.6 ± 7.22	<0.001
Weight, kg	58.5 ± 10.4	65.1 ± 11.0	69.2 ± 11.2	71.3 ± 12.1	74.7 ± 12.3	<0.001
BMI, kg/m^2	22.7 ± 3.31	23.9 ± 3.22	24.8 ± 3.30	25.1 ± 3.52	25.9 ± 3.54	<0.001
SBP, mmHg	117.5 ± 16.9	122.0 ± 16.7	124.3 ± 16.7	125.1 ± 16.2	128.1 ± 17.4	<0.001
DBP, mmHg	71.5 ± 10.3	74.8 ± 10.6	76.7 ± 10.3	77.6 ± 10.0	79.7 ± 11.4	<0.001
HR, per min	74.8 ± 10.1	74.1 ± 10.4	74.4 ± 10.4	73.9 ± 9.85	74.8 ± 10.4	0.87
NT-proBNP, ng/L	45.1 ± 42.3	47.7 ± 108	42.8 ± 136	39.1 ± 54.0	44.1 ± 113	0.27
HbA1c, mg/dL	5.67 ± 0.62	5.76 ± 0.84	5.83 ± 0.81	5.90 ± 0.99	6.02 ± 1.07	<0.001
Fasting glucose, mg/dL	95.5 ± 15.1	99.1 ± 19.8	102.1 ± 20.7	103.3 ± 25.3	106.7 ± 28.4	<0.001
Cholesterol, mg/dL	198.3 ± 36.3	200.3 ± 40.2	203.4 ± 35.4	203.3 ± 37.3	201.5 ± 35.7	0.01
Triglyceride, mg/dL	114.8 ± 105.9	133.2 ± 144.5	141.8 ± 90.9	144.1 ± 89.3	159.5 ± 115.2	<0.001
LDL, mg/dL	123.6 ± 33.3	128.4 ± 34.0	132.4 ± 31.1	133.4 ± 33.8	132.2 ± 32.7	<0.001
HDL, mg/dL	59.1 ± 16.0	54.0 ± 14.5	51.8 ± 14.2	50.6 ± 13.7	48.4 ± 12.8	<0.001
eGFR, mL/min/1.73m^2	94.1 ± 18.8	89.3 ± 17.0	87.315.3	86.7 ± 16.7	84.5 ± 16.3	<0.001
QRS duration, ms	85.9 ± 10.0	88.6 ± 10.3	90.0 ± 11.6	91.3 ± 11.1	93.4 ± 12.1	<0.001
Hypertension, %	187 (12.7%)	146 (14.9%)	164 (17%)	173 (20.7%)	287 (27.0%	<0.001
Diabetes, %	68 (4.6%)	50 (5.1%)	64 (6.6%)	59 (7.1%)	103 (9.7%)	<0.001
Hyperlipidemia, %	100 (6.8%)	69 (7.0%)	70 (7.2%)	78 (9.3%)	93 (8.7%)	0.02
CAD, %	77 (5.2%)	58 (5.9%)	52 (5.4%)	48 (5.7%)	76 (7.1%)	0.08

Alcohol, %	54 (3.7%)	48 (4.9%)	70 (7.2%)	66 (7.9%)	101 (9.5%)	<0.001
Exercise, %	216 (14.6%)	137 (14.0%)	135 (14.0%)	120 (14.4%)	157 (14.8%)	0.91
Smoking, %	4 (0.3%)	8 (0.8%)	4 (0.4%)	6 (0.7%)	15 (1.4%)	0.003

AoD: aortic root diameter; BMI: body mass index; SBP: systolic blood pressure; DBP: diastolic blood pressure; HR: heart rate; Hb: hemoglobin; WBC count: circulating white blood cell count; LDL: low-density lipoprotein; HDL: high-density lipoprotein; GPT: glutamic-pyruvate transaminase; eGFR: estimated glomerular filtration rate; CAD: coronary artery disease.

Table S2. NT-proBNP level and echocardiographic parameters stratified by aortic root diameter

AoD Quintiles	1st Quintile (*n* = 1481)	2nd Quintile (*n*=982)	3rd Quintile (*n* = 968)	4th Quintile (*n* = 1161)	5th Quintile (*n* = 748)	*p* (Trend)	Pearson's Correlation	Coef	95% CI	*p* Value
NT-proBNP, ng/L	45.1 ± 42.3	47.7 ± 108	42.8 ± 136	39.1 ± 54.0	44.1 ± 113	0.27	-0.008	-1.84	-8.56, 4.89	0.59
IVS, mm	8.47 ± 1.09	8.90 ± 0.95	9.15 ± 0.96	9.31 ± 0.96	9.61 ± 1.02	<0.001	0.398	1.07	1.00, 1.13	<0.001
PWd, mm	8.48 ± 1.00	8.91 ± 0.89	9.14 ± 0.88	9.36 ± 1.41	9.61 ± 0.89	<0.001	0l395	1.06	1.00, 1.13	<0.001
LVIDd, mm	44.3 ± 3.44	46.2 ± 3.29	47.3 ± 3.12	47.8 ± 3.35	48.6 ± 3.27	<0.001	0.432	3.91	3.69, 4.12	<0.001
LVMI, g/m^2	70.0 ± 13.9	75.3 ± 13.4	77.8 ± 13.2	79.5 ± 13.4	83.2 ± 14.8	<0.001	0.348	12.7	11.7, 13.6	<0.001
RWT	38.6 ± 4.9	38.9 ± 4.3	39.1 ± 4.3	39.3 ± 4.5	39.9 ± 4.4	<0.001	0.11	0.13	0.10, 0.16	<0.001
LVH (%)	55 (3.9%)	38 (4.2%)	32 (3.7%)	26 (3.4%)	40 (4.2%)	0.89	—	—	—	—
EDVi	39.0 ± 6.3	40.5 ± 6.1	40.9 ± 6.0	41.4 ± 6.5	42.0 ± 6.7	<0.001	0.18	0.29	0.24, 0.33	<0.001
LVEF, %	63.2 ± 5.7	63.0 ± 5.0	62.8 ± 5.1	62.1 ± 5.4	62.0 ± 5.7	<0.001	-0.07	-0.10	-0.13, -0.06	<0.001
DT, ms	195.3 ± 42.4	200.7 ± 41.3	201.5 ± 39.8	206.6 ± 40.3	211.0 ± 45.3	<0.001	0.136	14.1	11.3, 16.9	<0.001
IVRT, ms	85.8 ± 13.4	87.9 ± 13.2	89.3 ± 13.5	91.0 ± 15.1	94.7 ± 17.8	<0.001	0.225	8.26	7.17, 9.35	<0.001
E/A ratio	1.33 ± 0.47	1.23 ± 0.41	1.20 ± 0.38	1.15 ± 0.41	1.07 ± 0.37	<0.001	-0.222	-0.23	-0.26, -0.20	<0.001
TDI-s′ (average), mm	8.24 ± 1.57	8.29 ± 1.53	8.37 ± 1.50	8.30 ± 1.59	8.24 ± 1.59	0.95	0.003	0.01	-0.09, 0.11	0.85
LAVi, mL/m^2	16.0 ± 5.53	16.1 ± 5.81	16.8 ± 5.93	16.3 ± 6.22	17.0 ± 5.88	<0.001	0.064	0.94	0.52, 1.35	<0.001
TDI-e′ (average), mm	9.98 ± 2.56	9.41 ± 2.32	9.18 ± 2.25	8.76 ± 2.26	8.23 ± 2.13	<0.001	-0.271	-1.61	-1.76, -1.45	<0.001
E/e′ (average)	7.73 ± 2.42	7.72 ± 2.34	7.86 ± 2.58	7.87 ± 2.53	8.08 ± 2.64	<0.001	0.056	0.35	0.18, 0.51	<0.001
TR velocity, m/sec	18.0 ± 5.27	18.0 ± 5.26	17.5 ± 5.44	17.6 ± 5.31	17.7 ± 5.51	0.06	-0.023	-0.30	-0.67, 0.07	0.11

AoD: aortic root diameter; ASE, American Society of Echocardiography; DT: mitral inflow deceleration time; E: early mitral inflow velocity; TDI-e′: peak mitral annulus relaxation velocity e′; E/A: mitral inflow E/A ratio; E/e′ mean: mean early mitral inflow E to mitral annulus relaxation velocity e′ ratio; IVRT: isovolumic relaxation time; IVS: interventricular septal wall thickness; LA: left atrial/atrium; LAVi: left atrial volume index; LVIDd: LV end-diastolic diameter; LVMI: left ventricular mass index; TDI-s′: peak mitral annulus systolic velocity; TR: tricuspid regurgitation; CI: confidence interval.
